# End of life care in a secure hospital setting

**DOI:** 10.1192/bjo.2021.288

**Published:** 2021-06-18

**Authors:** Owen Obasohan, Deepak Tokas, Mamta Kumari

**Affiliations:** TEWV NHS Foundation Trust

## Abstract

**Aims:**

To measure the standard of care provided to patients who had a natural and expected death whilst in secure care at Roseberry Park Hospital, Middlesbrough.

Mallard ward is a low secure psychiatric ward for older aged men suffering from cognitive difficulties and significant physical comorbidity in addition to a severe and enduring mental illness. The patient population is such that it will remain the most appropriate placement for some patients until their death. It is vital that staff members on Mallard ward and indeed all parts of the Trust are aware of the priorities for care of the dying person and ensure that care is provided in accordance with these priorities.

The Leadership Alliance for the Care of Dying People (LACDP), a coalition of 21 national organisations, published One Chance to get it Right – Improving people's experience of care in the last few days and hours of life in June 2014. This document laid out five priorities for care of the dying person focussing on sensitive communication, involvement of the person and relevant others in decisions and compassionately delivering an individualised care plan.

**Method:**

The data collection tool was adapted from End of Life Care Audit: Dying in Hospital, a national clinical audit commissioned by Healthcare Quality Improvement Partnership (HQIP) and run by the Royal College of Physicians. Data were collected from both electronic and paper records. There were three natural and expected deaths in the last two years.

**Result:**

For all three patients, there was documented evidence that they were likely to die in the coming hours or days.

End of life care discussion was held with the nominated persons and not with the patients due to their lack of mental capacity.

The needs of the patients and their nominated persons were explored in all three cases.

All patients had an individualised care plan which was followed.

The palliative care team supported the staff with the care of these patients.

The care provided was largely consistent with the priorities listed.

**Conclusion:**

The national audit compares performance of only acute NHS Trusts with no data to reflect the performance of mental health hospitals. It is imperative that mental health services work in collaboration with physical health and palliative care services so they are able to continue providing a high level of care to this patient group. Clinicians and staff involved in the care of dying patients also need to be adequately trained.

